# Knowledge and Attitudes Toward Trachoma Among Final‐Year Clinical Medicine Students in Dar es Salaam, Tanzania: A Descriptive Cross‐Sectional Study

**DOI:** 10.1155/jotm/8823107

**Published:** 2026-07-29

**Authors:** Laurent Elisaut Marishamu, Irene Masuki, Heveanlight Masuki, Vivian P. Mushi, Celina Mhina, Ntsilane Suzan Mosenene, Milka Mafwiri

**Affiliations:** ^1^ Department of Ophthalmology, Muhimbili University of Health and Allied Sciences, Dar es Salaam, Tanzania, muchs.ac.tz; ^2^ Department of Clinical Medicine, St. Bakhita Health Training Institute, Namanyere, Nkasi, Tanzania; ^3^ Department of Parasitology and Medical Entomology, Muhimbili University of Health and Allied Sciences, Dar es Salaam, Tanzania, muchs.ac.tz; ^4^ Department of Zoology and Wildlife Conservation, University of Dar es Salaam, Dar es Salaam, Tanzania, udsm.ac.tz

**Keywords:** attitudes, knowledge, medical students, Tanzania, trachoma

## Abstract

**Background:**

Trachoma continues to pose a serious concern as one of the leading causes of blindness globally, particularly in areas with poor access to healthcare and hygiene services. Despite its importance, there is a conspicuous paucity of thorough data on medical students’ awareness and attitudes toward trachoma. To fill this gap, a study was done to investigate the knowledge and attitudes toward trachoma among final‐year clinical medicine students at Dar es Salaam Colleges, shedding light on the awareness levels and attitudes of this key group of future healthcare practitioners.

**Methods:**

A descriptive cross‐sectional study was conducted at three colleges, with 384 individuals recruited using simple random selection for a questionnaire survey. Self‐administered questionnaires were used to assess the knowledge and attitudes of trachoma among final‐year clinical medicine students in Dar es Salaam colleges. The collected data were analyzed using descriptive statistics and the chi‐square test to summarize the students’ knowledge and attitudes toward trachoma. A *p* value of less than 0.05 was considered statistically significant.

**Results:**

The study revealed that nearly half of the participants (49%) had a low level of knowledge about trachoma prevention, while approximately 43% held negative attitudes toward it. Significant associations were found between knowledge level and age (*p* = 0.037) as well as sex (*p* = 0.039). Notably, females and individuals aged 25–29 years demonstrated lower levels of knowledge.

**Conclusion and Recommendations:**

A significant number of medical students had low knowledge about trachoma and harbored negative attitudes toward its prevention. Consequently, ongoing education and training are essential to enhance understanding among current students, alongside incorporating case studies for practical application.

## 1. Introduction

Trachoma is among the neglected tropical diseases (NTDs) that are more prevalent in low‐income settings [1, 2]. It is a preventable and treatable disease and the most common infectious cause of blindness worldwide [3]. It is caused by the bacterium *Chlamydia trachomatis* and remains a significant public health concern [4]. Globally, the number of people at risk of trachoma was 142.2 million, and it was responsible for the visual impairment of about 1.9 million people in 2019 [1]. It is estimated that about 182 million people live in trachoma‐endemic areas in 42 countries [2, 4]. Of all continents, Africa is among the most highly affected and requires the most intensive control efforts [3, 5].

Tanzania, like many other countries in SSA, has failed to eliminate trachoma [5] and currently has a prevalence of 25.4% in trachoma‐endemic areas, and an additional 12.5 million people are at risk of trachoma [6]. Known risk factors include lack of water, poor personal hygiene at an individual, family, or community level, age and sex; common in children and women, lack of education and understanding about the spread of contagious diseases [7], and environmental sanitation, considered environmental factors known as the four Ds (dust, dry, dirty, and discharge) [1, 8].

To achieve the elimination of the disease, different countries adopted the World Health Organization Surgery, Antibiotic, Facial Cleanliness, and Environmental Improvement (WHO‐SAFE) for Trachoma control [2, 9, 10]. Tanzania’s Ministry of Health (MOH) has adopted SAFE strategies and has implemented them in eight districts across three regions [6]. Despite this effort, trachoma remains one of the major health problems in the country. Studies conducted in Kenya and Ethiopia show that poor knowledge and unfavorable attitudes toward trachoma had an impact on the transmission and control of the disease, leading to an increased prevalence of the disease in the community [1–3, 9].

Medical practitioners are the ones expected to provide health education to communities concerning the disease. Currently, there is limited data on the knowledge and attitudes toward trachoma among medical students. Therefore, a study to assess the knowledge and attitude toward trachoma among diploma clinical medicine students was conducted. This cadre was selected because most of the practitioners working at the lower‐level health facilities across the country are clinical officers, clinical assistants, and nonprofessional medical staff. These practitioners operate directly within rural communities, serving as the initial point of contact for patients and delivering essential health education.

## 2. Methods

### 2.1. Study Setting

The study was conducted in three selected colleges of health sciences in the Dar es Salaam region, Tanzania. According to the list of colleges registered in the National Council for Technical and Vocational Education and Training (NACTVET) academic year 2019/20, the total number of colleges of health that offer diplomas in clinical medicine was 15, and only 2 were owned by the government. The distribution of these colleges was as follows: Ilala District had 3 colleges, Kinondoni District had 5, Kigamboni District had 2, Temeke District had 3, and Ubungo District had 2. Each college enrolled approximately 200 or more students per intake. There were two intakes annually, in March and September.

### 2.2. Study Design

A descriptive cross‐sectional study utilizing a quantitative method of data collection was conducted from October to November 2021 in the Dar es Salaam region to assess knowledge and attitudes toward trachoma among final‐year clinical medicine students at the College of Health and Allied Sciences in Dar es Salaam.

### 2.3. Study Population and Eligibility Criteria

The study population was final‐year clinical medicine students in three selected colleges of Health and Allied Sciences in Dar es Salaam. Only students who were available during the day of data collection and signed the consent form were eligible for the study. Those who were absent on the day of data collection were excluded from the study.

### 2.4. Sample Size and Sampling Technique

The sample size for this study was calculated using the cross‐sectional formula: *n* = *Z* ^2^ *p*(100 − *p*)/*ε* ^2^. In this formula, *Z* is the standard normal deviation (1.96) for a 95% confidence interval, *p* is the estimated population proportion (51%), and *ε* ^2^ represents the margin of error, set at approximately 5%. To account for potential nonresponses, a 10% adjustment was included, yielding a final sample size of 422 participants [1].

A two‐stage random sampling technique was employed for this study. In the first stage, three districts (Temeke, Kigamboni, and Ubungo) were selected randomly from the five districts in Dar es Salaam. In the second stage, one college was randomly selected from each of the selected districts.

**FIGURE 1:**
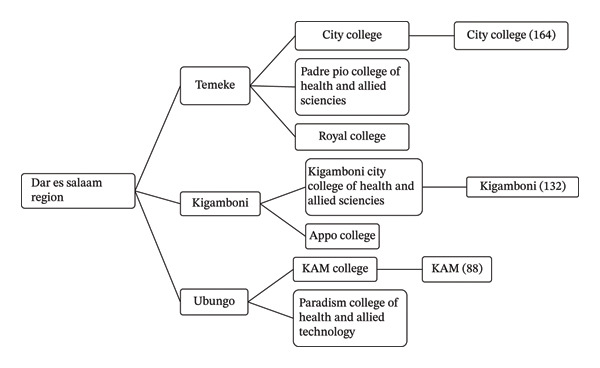
Flowchart showing sampling procedures.

Within the college, a census approach was employed, whereby all registered final‐year students who were present on the day of data collection were invited to participate. Of the 422 students approached, 384 consented to participate, yielding a response rate of 91%. Following data cleaning, 13 questionnaires were excluded due to missing primary outcome data, leaving 371 participants for the final analysis of knowledge and attitudes (Figure 1).

### 2.5. Questionnaire Survey

A self‐constructed questionnaire was used as the primary tool for data collection. The questionnaire comprised three sections: Section A focused on the demographic profiles of the participants, while Section B inquired about their understanding of trachoma. Section C explored the attitudes of the respondents toward trachoma (Supporting File 1). Information regarding the clinical characteristics, transmission, treatment, and prevention methods associated with trachoma was solicited. The questionnaire predominantly consisted of closed‐ended questions, with one open‐ended query included. The questionnaire was developed in English and self‐administered by the participants.

### 2.6. Data Collection Procedure

Participants were informed about the study. All eligible participants in each college were requested to participate. All participants signed the consent form on the day of the study, before the distribution of questionnaires. Participants were instructed on how to complete the questionnaires.

### 2.7. Outcome and Independent Variables

The outcome of the study was knowledge levels and attitudes toward trachoma. Knowledge was reported at high, moderate, and low levels of knowledge, and attitudes were reported as positive and negative. The independent variables were age, sex (male and female), previous education level (ordinary level, advanced level, and certificate in clinical medicine), marital status (single, married/cohabiting, separated, or widow/widower), and college (City College of Health and Allied Sciences [CCoHAS], Kigamboni City College of Health and Allied Sciences [KiCCoHAS] and KAM College of Health).

### 2.8. Quality Control

The quality of the study was ensured by pretesting the questionnaire at a college in the Kinondoni District, Dar es Salaam, using 10% of the intended sample size before data collection. Following the pilot test, minor wording revisions were made to the transmission section of the questionnaire to improve clarity. A semistructured questionnaire comprising both closed and open‐ended questions was initially developed in English and was not translated, as English is the language of instruction and examination for clinical medicine programs in Tanzania. Data were collected by the principal investigator under the supervision of experienced supervisors who monitored the data collection process.

### 2.9. Data Management and Analysis

The data were entered and cleaned through EpiData Version 3 (https://www.epidata.dk) and exported to the Statistical Package for Social Sciences (SPSS) Version 28.0 (IBM Corp., Armonk, NY, USA) for statistical analysis. The data were analyzed for each specific objective using descriptive statistics, reported as proportions. The chi‐square test was employed to compare these proportions.

Knowledge was assessed using a scoring scale comprising 10 questions about trachoma infection, with a maximum possible score of 19. Although the questionnaire contained 10 questions, some items allowed multiple responses. For example, participants could identify up to three transmission routes, three risk factors, three symptoms/signs, and the four components of the SAFE strategy. Consequently, the maximum attainable knowledge score was 19. Each correct answer was assigned a score of one, while incorrect answers received zero points. Participants scoring ≥ 15 (≥ 75%) of 19 were classified as having high knowledge. Those scoring between 9 and 14 (50%–74%) were categorized as having medium knowledge. Conversely, participants scoring below nine (50% or less) were classified as having low knowledge. Attitudes were assessed using a five‐point Likert scale, consisting of 10 statements, each rated from 1–5 points, with a Cronbach’s alpha value of 0.633. A total score was then calculated, resulting in a scale ranging from 10 to 50 points. The mean attitude score was used to classify attitudes as either positive or negative. Positive attitudes scored ≥ 44 points or higher, while negative attitudes scored ≤ 43 points or lower. The association between demographic characteristics and knowledge and attitudes toward trachoma was assessed using Pearson’s chi‐square test. A *p* value of less than 0.05 was considered statistically significant.

### 2.10. Ethical Consideration

The proposal was submitted to the Institutional Review Board of MUHAS with Ref No. DA.25/111/01B and Permit letters were submitted to KAM College, KiCCoHAS, and CCoHAS. Students were informed about the study and requested to participate. Participation was voluntary, and informed consent was obtained before data collection. Confidentiality and privacy were observed throughout the research and even after the research. The information that may lead to the identification of participants, such as names, phone numbers, or emails, was not included in the questionnaire. Storage of the questionnaire after completion of the research activity was in archives in such a way that unauthorized people did not have access to it.

## 3. Result

### 3.1. Sociodemographic Characteristics of the Participants

A total of 384 individuals met the inclusion criteria, resulting in a response rate of 91%. Over half of the participants (52.7%) were male, with the majority falling within the 18–24 age range, and approximately 42.7% were affiliated with CCoHAS (Table 1).

**TABLE 1:** Sociodemographic characteristics of the participants (*N* = 384).

Variable	Category	*n*	%
Age	18–24	281	73.2
	25–29	88	22.9
	30+	15	3.9

Sex	Male	201	52.3
	Female	183	47.7

Marital status	Married	30	7.8
	Single	337	87.8
	Separated/divorced	3	0.8
	Cohabiting	14	3.6
	Widow/widowed	0	0.0

Education level	Ordinal level	50	13.0
	Advance level	68	17.7
	Certificate	266	69.3

College	CCOHAS	164	42.7
	KiCCOHAS	132	34.4
	KAM	88	22.9

*Note:* KiCCoHAS = Kigamboni City College of Health and Allied Sciences, and KAM College of Health.

Abbreviation: CCoHAS, City College of Health and Allied Sciences.

### 3.2. Knowledge of Trachoma Among Final‐Year Clinical Medicine Students

The vast majority (96.6%) had heard about trachoma, with 275 (74.1%) identifying trachoma as an eye disease caused by bacteria. The most mentioned symptom of trachoma was red eyes (79.5%). Regarding disease control, 317 (85.4) and 268 (72.2%) correctly responded that environmental control and face washing are the means of controlling the disease, respectively (Table 2).

**TABLE 2:** Knowledge on trachoma among final‐year clinical medicine students.

Variable	Categories	Frequency (*n*)	Percentage (%)
Awareness of trachoma	Yes	371	96.6

Causes/definition of trachoma	A viral infection that affects the eye	59	15.9
	A bacterial infection that affects the eyes	275	74.1
	A fungal infection that affects the eyes	4	1.1
	An injury to the eyes	30	8.1
	I do not know	3	0.8

Signs/symptoms (multiple responses)	Red eyes	295	79.5
	Eye rash	59	15.9
	Watery eyes	108	29.1
	Poor eyesight	57	15.4

Risk group (multiple responses)	Children < 10 years	161	43.4
	Teens 13–17 years	14	3.8
	Adults (over 18 years)	15	4.0
	Everybody	185	49.9
	Older people	18	4.9
	I don’t know	7	1.9

Transmission (multiple responses)	Through flies	223	60.1
	By contaminated fingers	162	43.7
	By sharing contaminated clothes/towels	46	12.4
	I don’t know	4	1.1

Trachoma control (multiple responses)	Surgery	45	12.1
	Antibiotics	61	16.4
	Face	268	72.2
	Environmental	317	85.4

*Note:* Denominator was 371 of 384 due to 13 missing data points on the outcome.

### 3.3. Attitudes Toward Trachoma Among Final‐Year Clinical Medicine Students

Most of the respondents, 316 (85.2%), strongly agreed that trachoma is a treatable disease. Also, 289 (77.9%) and 280 (75.5%) of the respondents strongly agreed that trachoma is a preventable disease/problem and that face cleaning is good for preventing trachoma, respectively. However, 158 (42.6%) of the participants do not believe that sharing a towel is a good practice for trachoma prevention (Table 3).

**TABLE 3:** Attitudes toward trachoma among final‐year clinical medicine students.

Items for the attitude on trachoma	Strongly agree *n* (%)	Agree *n* (%)	Neutral *n* (%)	Disagree *n* (%)	Strongly disagree *n* (%)
Trachoma is a treatable disease.	316 (85.2)	50 (13.5)	5 (1.3)	0 (0)	0 (0.0)
Trachoma is a preventable disease/problem.	289 (77.9)	76 (20.5)	4 (1.1)	2 (0.5)	0 (0.0)
Surgical correction is important for the treatment of trachoma.	142 (38.3)	87 (23.5)	48 (12.9)	67 (18.1)	27 (7.3)
Face cleaning is good for preventing trachoma.	280 (75.5)	76 (20.5)	12 (3.2)	2 (0.5)	1 (0.3)
The use of latrines is beneficial for the prevention of trachoma	190 (51.2)	118 (31.8)	24 (6.5)	31 (8.4)	8 (2.2)
Control of house flies has a great benefit for the prevention of trachoma	253 (68.2)	86 (23.2)	16 (4.3)	13 (3.5)	3 (0.8)
Hand washing is always helpful for trachoma prevention	292 (78.7)	65 (17.5)	9 (2.4)	5 (1.3)	0 (0.0)
Taking antibiotics is often an important measure for trachoma prevention	182 (49.1)	100 (27.0)	29 (7.8)	43 (11.6)	17 (4.6)
Sharing a towel is a good practice for trachoma prevention	69 (18.6)	51 (13.7)	17 (4.6)	76 (20.5)	158 (42.6)
Blindness can occur due to trachoma	288 (77.6)	64 (17.3)	13 (3.5)	3 (0.8)	3 (0.8)

*Note:* Denominator is 371 of 384 due to 13 missing data points on the outcome.

### 3.4. Classification of Knowledge and Attitude Toward Trachoma Among Final Year Clinical Medicine Students

**FIGURE 2:**
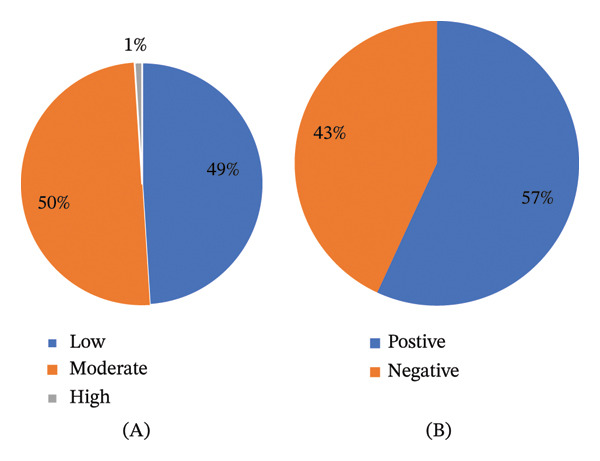
Categorization of trachoma knowledge levels among final‐year clinical medicine students (A) and classification of attitudes toward trachoma prevention among final‐year clinical medicine students (B).

Nearly half of the participants (49%) had poor knowledge of trachoma, as shown in Figure 2A. Additionally, around 43% had negative attitudes toward trachoma, as shown in Figure 2B.

### 3.5. Influence of the Social Demographic Characteristics on the Knowledge Toward Trachoma

A statistically significant association was observed between age group and knowledge of trachoma (*p* = 0.037). Participants aged 18–24 years and 25–29 years had the highest proportions of low knowledge, with 137 (49.8%) and 41 (50.6%) participants, respectively, classified as having low knowledge. Furthermore, significant differences in knowledge levels were observed between genders (*p* = 0.039), with a higher proportion of females having low knowledge of trachoma when compared to males (Table 4).

**TABLE 4:** Influence of the social demographic characteristics on the knowledge toward trachoma.

Sociodemographic characteristics		Knowledge of trachoma			*p* value
		High	Moderate	Low	
Age group	18–24	1 (0.4)	137 (49.8)	137 (49.8)	0.037^∗^
	25–29	1 (1.2)	39 (48.1)	41 (50.6)	
	30+	1 (6.8%)	7 (46.6%)	7 (46.6%)	

Sex	Male	2 (1.0)	110 (55.6)	86 (43.4)	0.039^∗^
	Female	1 (0.6)	74 (42.8)	98 (56.6)	

Marital status	Single	2 (0.6)	161 (49.5)	162 (49.8)	0.794
	Married	1 (3.3)	15 (50.0)	14 (46.7)	
	Cohabiting	0 (0.0)	6 (46.2)	7 (53.8)	
	Divorced/separated	0 (0.0)	2 (66.7)	1 (33.3)	

College name	CCOHAS	1 (0.6)	78 (48.1)	83 (51.2)	0.087
	KICCOHAS	1 (0.8)	57 (43.5)	73 (55.7)	
	KAM	1 (1.3)	49 (62.8)	28 (35.9)	

Previous education level	Certificate in clinical medicine	2 (0.8)	136 (52.7)	120 (46.5)	0.292
	Advanced secondary certificate	1 (1.5)	25 (38.5)	39 (60.0)	
	Ordinary secondary certificate	0 (0.0)	23 (47.9)	25 (52.1)	

*Note:* KiCCoHAS = Kigamboni City College of Health and Allied Sciences, and KAM College of Health. Denominator is 371 of 384 due to 13 missing outcome data.

Abbreviation: CCoHAS, City College of Health and Allied Sciences.

^∗^Statistically significant (*p* < 0.05).

### 3.6. Influence of the Social Demographic Characteristics on the Attitudes Toward Trachoma Prevention

The majority of participants across various demographic groups demonstrated positive attitudes toward trachoma prevention. However, none of the sociodemographic characteristics were statistically significantly associated with positive attitudes toward trachoma prevention among the study participants (Table 5).

**TABLE 5:** Influence of the sociodemographic characteristics on attitudes toward trachoma prevention.

Sociodemographic characteristics		Attitude		*p* value
		Positive *n* (%)	Negative *n* (%)	
Age group	18–24	153 (55.6)	122 (44.4)	0.733
	25–29	49 (60.5)	32 (39.5)	
	30+	6 (54.5)	5 (45.5)	

Sex	Male	117 (59.1)	81 (40.9)	0.251
	Female	94 (54.3)	79 (45.7)	

Marital status	Single	185 (56.9)	140 (43.1)	0.489
	Married	19 (63.3)	11 (36.7)	
	Cohabiting	5 (38.5)	8 (61.5)	
	Divorced/separated	2 (66.7)	1 (33.3)	

College name	CCOHAS	94 (58.0)	68 (42.0)	0.080
	KICCOHAS	81 (61.8)	50 (38.2)	
	KAM	36 (46.2)	42 (53.8)	

Previous education level	Clinical medicine certificate	152 (58.9)	106 (41.1)	0.144
	Advanced secondary certificate	38 (58.5)	27 (41.5)	
	Ordinary secondary certificate	21 (43.8)	27 (56.2)	

*Note:* KiCCoHAS = Kigamboni City College of Health and Allied Sciences, and KAM College of Health. Denominator is 371 of 384 due to 13 missing outcome data.

Abbreviation: CCoHAS, City College of Health and Allied Sciences.

## 4. Discussion

This study aimed to assess knowledge and attitudes toward trachoma among final‐year clinical medicine students in Dar es Salaam. The majority (96.6%) of students were aware of trachoma, likely due to the endemic nature of the disease and their background as medical students. This finding is consistent with studies conducted in northern and southern Ethiopia, where over 90% of the community was aware of the disease [1, 10]. This suggests that trachoma is widespread in the area, affecting a large population, and its consistent presence contributes to increased awareness. Such awareness can play a crucial role in facilitating student understanding and promoting effective disease‐control efforts.

We observed that a significant number of students had inadequate knowledge (49%) and negative attitudes (43%) toward trachoma control and prevention. Trachoma is among the NTDs in Tanzania [1, 5], and most of the curriculum in our setting is not well emphasized, thus leading to low knowledge about the disease. Students with positive attitudes may be influenced by their residence in an area endemic to infectious diseases and their enrollment in medical studies, whereas negative attitudes could stem from a lack of knowledge about the subject.

In this study, the majority of the respondents were familiar with trachoma, likely due to the endemic nature of the disease in Tanzania. These findings align closely with studies conducted in communities in Northern Ethiopia (89.2%), Southern Ethiopia (92.6%), and Bangladesh (86%) [1, 11, 12]. In this study, 25.9% of participants were unaware that trachoma is caused by a bacterial infection that affects the eyes and can be transmitted from person to person. This lack of understanding can lead to misdiagnosis and improper management of the disease within the community. We observed that 12.4%, 43.7%, and 60% of students were aware that trachoma can be transmitted through contaminated clothes, fingers, and flies, respectively. This awareness level is significantly higher than that in a study conducted in Tigray, Ethiopia. However, it aligns closely with findings on the mode of transmission from other studies conducted in Southern Ethiopia and Kenya [2, 10, 12]. Understanding the correct transmission methods may arise from living in a country where Trachoma is endemic, witnessing family members and friends acquire the disease, and observing patients during clinical rotations at hospitals and clinics.

The negative attitudes observed among a group of medical students toward trachoma could affect the community’s acceptance of the Neglected Tropical Diseases Control Program for trachoma. As future healthcare providers are expected to guide trachoma patients and the community, their negative attitudes might influence perceptions of preventive measures among patients and the community at large. Regarding the group of students who had positive attitudes toward trachoma being a treatable and preventable disease, they also acknowledge that facial cleanliness is effective in preventing trachoma and that controlling house flies offers significant benefits in trachoma prevention. This positivity may be due to their medical training and understanding of various infectious diseases, which contribute to their favorable attitudes. These findings suggest that further education on trachoma and increased emphasis on the disease during their studies could have a positive impact. Similar findings have been observed in studies conducted in other trachoma‐endemic countries [3].

### 4.1. Study Limitations

• A cross‐sectional study design was employed, capturing data at a single point in time. Consequently, causal relationships between variables cannot be inferred, and changes in knowledge and attitudes over time cannot be assessed.
• The study was conducted in three colleges within one region, limiting the generalizability of the findings to all final‐year clinical medicine students in other regions. Variations in culture, health resources, and curriculum changes could influence knowledge and attitudes toward trachoma differently in other settings.

## 5. Conclusions and Recommendations

The study investigated the knowledge and attitudes toward trachoma among final‐year clinical medicine students, revealing crucial insights that can inform future interventions and public health strategies. Despite a significant number of students being aware of trachoma, many had low levels of knowledge and negative attitudes toward trachoma prevention. This underlines the crucial need for ongoing education and training on trachoma for current medical students. Integrating case studies and practical experiences into the curriculum could greatly enhance students’ understanding and empathy for trachoma patients, improving their ability to diagnose and manage the condition effectively in their future practices. Finally, enhancing knowledge is likely to shift negative attitudes toward trachoma prevention.

**Nomenclature**
MoH: Ministry of Health
NACTVET: National Council for Technical and Vocational Education and Training
NTD: Neglected Tropical Disease
SAFE: Surgery, Antibiotics, Facial cleanliness, and Environmental sanitation
WHO: World Health Organization
NTDCP: Neglected Tropical Disease Control Program

## Author Contributions

Laurent Elisaut Marishamu conceived the study, designed the methodology, conducted the analysis, and wrote the manuscript. Milka Mafwiri provided ongoing supervision throughout the study. Heveanlight Masuki, as a co‐supervisor, contributed to the analysis and manuscript preparation. Irene Masuki participated in manuscript writing, Celina Mhina contributed to the methodology, Ntsilane Suzan Mosenene handled the introduction and data analysis, and Vivian P. Mushi critically reviewed and edited the manuscript.

## Funding

No funding was received for this research.

## Ethics Statement

The proposal was submitted to the IRB of MUHAS, and permit letters were obtained from KAM College, KiCCoHAS, and CCoHAS. Students were informed about the study and invited to participate voluntarily. Before data collection, informed consent was obtained from all participants. Confidentiality and privacy were strictly maintained throughout and beyond the data collection. The questionnaire excluded information identifying participants, such as names, phone numbers, or emails. Questionnaire storage after completion of the research was in secure archives to prevent unauthorized access.

## Conflicts of Interest

The authors declare no conflicts of interest.

## Supporting Information

Additional supporting information can be found online in the Supporting Information section.

## Supporting information


**Supporting Information** Supporting File 1: Questionnaire used for data collection (English).

## Data Availability

Data are available on request from the authors.
