# Supplementary material for: Knowledge and Attitudes Toward Trachoma Among Final‐Year Clinical Medicine Students in Dar es Salaam, Tanzania: A Descriptive Cross‐Sectional Study
Source: J Trop Med. 2026 Jul 29;2026:8823107. doi: 10.1155/jotm/8823107 (PMC13420301; doi:10.1155/jotm/8823107)
Supplement: Supplementary file 1 — Supporting Information Supporting File 1: Questionnaire used for data collection (English). [file JOTM-2026-8823107-s001.docx]

## ENGLISH QUESTIONNAIRE

Dear participant,

This questionnaire is for collecting information about knowledge and attitude of trachoma among final year students of clinical medicine in Dar Es Salaam colleges. Do not put your name on it and the answers will be confidential. Please, answer by putting a circle at alternative choice for each question and/or by writing your answer on the line beside the question, some questions will have instructions. Your participation is highly appreciated.

COLLEGENAME……………………………

SECTION A: SOCIODEMOGRAPHIC DATA

1. Sex
2. Male
3. Female
4. How old are you? ...........
5. What is your marital status?
   1. Single
   2. Married
   3. Cohabiting
   4. Divorced or separated
   5. Widowed
6. Your Level of education before joining the college
7. Ordinary secondary level
8. Advance secondary level
9. Certificate in clinical medicine level

**SECTION B: ASSESSMENT OF KNOWLEDGE TOWARD TRACHOMA**

1. Have you ever heard of trachoma?
2. Yes
3. No

(if no end the questionnaire)

1. Where did you hear about trachoma?

(Multiple responses are allowed)

1. From college
2. From Family
3. From Health facility
4. Friend
5. From written document
6. What is trachoma?
7. A viral infection that affects the eyes
8. A bacterial infection that affects the eyes
9. A fungal infection that affects the eyes
10. An injury of the eyes
11. I don’t know
12. What are the main symptoms for trachoma? (multiple response allowed)
13. Red eyes
14. Eye rash
15. Watery eyes
16. Poor eye sight
17. Others……
18. Can blindness occur due to trachoma?
19. Yes
20. No
21. I don’t know
22. Which age groups are mostly affected by trachoma? (multiple response allowed)
23. Children<10years
24. Teens 13-17 years
25. Adults (over 18 years)
26. Everybody
27. Older people
28. I Don’t know
29. Is trachoma a transmissible disease?
30. Yes
31. No
32. I don’t know

(If yes go to question 13, 1f no go questions 14)

1. How is the disease transmitted from infected person to non-infected person?
2. Through flies
3. By contaminated fingers
4. By sharing contaminated clothes / towels
5. I don’t know
6. Is ‘proper utilization of latrine’ a method to prevent transmission of Trachoma?
7. Yes
8. No
9. I don’t know
10. Is trachoma treatable?
11. Yes
12. No
13. I don’t know
14. Does ‘treating trachoma cases’ serve as a method to prevent transmission of Trachoma?
15. Yes
16. No
17. I don’t know
18. Mention four ways of how to prevent trachoma
19. ……………………………….………………………………………………….
20. .............................................................................................................................
21. ..............................................................................................................................
22. ..................................................................................................................

**SECTION C: ATTITUDE TOWARD TRACHOMA**

In this section put **(˅)** on provided table at response part

| **S/N** | **QUESTION** | **RESPONSE** | | | | |
| --- | --- | --- | --- | --- | --- | --- |
|  |  | **STRONGLY AGREE** | **AGREE** | **NEUTRAL** | **DISAGREE** | **STRONGLY DISAGREE** |
|  | Trachoma is a treatable disease. |  |  |  |  |  |
|  | Trachoma is a preventable diseases/problem. |  |  |  |  |  |
|  | Surgical correction is important for the treatment of trachoma. |  |  |  |  |  |
|  | Face cleaning is good for preventing trachoma. |  |  |  |  |  |
|  | The use of latrines is beneficial for prevention of trachoma |  |  |  |  |  |
|  | Control of house flies has great benefit for prevention of trachoma |  |  |  |  |  |
|  | Hand washing is always helpful for trachoma prevention |  |  |  |  |  |
|  | Taking antibiotics is often an important measure for trachoma prevention |  |  |  |  |  |
|  | Do you think that sharing a towel is a good practice for trachoma prevention? |  |  |  |  |  |
|  | Blindness can occur due to trachoma |  |  |  |  |  |

***THANK YOU FOR YOUR PARTICIPATION!!***
